# Supplementary material for: Psychological Distress, Dental Health, and Dental Fear among Finnish University Students: A National Survey
Source: Int J Environ Res Public Health. 2021 Sep 29;18(19):10245. doi: 10.3390/ijerph181910245 (PMC8508373; doi:10.3390/ijerph181910245)
Supplement: Supplementary file 1 [file ijerph-18-10245-s001.zip › ijerph-1389781-supplementary.pdf]

Table S1. Variables Used in the Study with the Question(s), Answer Options and Dicotomization.

Variable	Question(s)	Answer options	Dicotomization
Dental fear	"Do you feel scared about receiving dental care?"	1= "Not at all"<br>2= "Somewhat"<br>3= "Very"	0= Not at all or somewhat<br>1= Very
Clinical Outcomes in Routine Evaluation 10 (CORE-10)	(1) "I have felt tense, anxious or nervous"<br>(2) "I have felt I have someone to turn to for support when needed"<br>(3) "I have felt able to cope when things go wrong"<br>(4) "Talking to people has felt too much for me"<br>(5) "I have felt panic or terror"<br>(6) "I made plans to end my life"<br>(7) "I have difficulty getting to sleep or staying asleep"<br>(8) "I have felt despairing or hopeless"<br>(9) "I have felt unhappy"<br>(10) "Unwanted images or memories have been distressing me".	5-point scale<br>0= "Not at all"<br>1= "Only occasionally"<br>2= "Sometimes"<br>3= "Often"<br>4= "Most or all the time"	0= Scores ≤ 10 representing a lower or negative CORE-10 score and scores<br>1= Scores ≥ 11 representing higher or positive CORE-10 score
General Health Questionnaire 12 (GHQ-12)	(1) "Have you recently been able to concentrate on your tasks?"	Four-point scale with bi-modal (0-0-1-1)<br>0= "Better than usual"<br>0= "Same as usual"<br>1= "Less than usual"<br>1= "Much less than usual"	0= Scores ≤ 3 representing a lower or negative GHQ-12 score<br>1= Scores ≥ 4 representing a higher or positive GHQ-12 score
	(2) "Have you recently lost much sleep because you've been worried?"	0= "Not at all"<br>0= "No more than usual"<br>1= "Rather more than usual"<br>1= "Much more than usual"	
	(3) "Have you recently felt that you are playing a useful part in things?"	0="More than usual"<br>0= "Same as usual"<br>1="Less useful than usual"<br>1= "Much less than usual"	
	(4) "Have you recently felt capable of making decisions"	0= "Better than usual"<br>0= "Same as usual"<br>1= "Less than usual"	

	1= "Much less than usual")<br>0= "Not at all"
(5) "Have you recently felt constantly under strain?"	0= "No more than usual"<br>1= "Rather more than usual"<br>1= "Much more than usual"
(6) "Have you recently felt that you could not overcome your difficulties?"	0= "Not at all"<br>0= "No more than usual"<br>1= "Rather more than usual"<br>1= "Much more than usual"
(7) "Have you recently been able to enjoy your day-to-day activities?"	0= "More than usual"<br>0= "Same as usual"<br>1= "Less than usual"<br>1 = "Much less than usual"
(8) "Have you recently been able to face up to your problems?"	0= "Better than usual"<br>0= "Same as usual"<br>1= "Less than usual"<br>1= "Much less than usual"
(9) "Have you recently been feeling unhappy and depressed?"	0= "Not at all"<br>0= "No more than usual"<br>1= "Rather more than usual"<br>1= "Much more than usual"
(10) "Have you recently been losing confidence in yourself?"	0= "Not at all"<br>0= "No more than usual"<br>1= "Rather more than usual"<br>1= "Much more than usual"
(11) "Have you recently been thinking of yourself as a worthless person?"	0= "Not at all"<br>0= "No more than usual"<br>1= "Rather more than usual"<br>1= "Much more than usual"
(12) "Have you recently been feeling reasonably happy, all things considered?"	0= "More so than usual"<br>0= "Same as usual"

		1= "Less so than usual"<br>1= "Much less than usual	
Teeth-related symptoms	"Have you had tooth problems (shooting pain, toothache) over the past month (30 days)?"	1= "Not at all"<br>2= "Every now and then"<br>3= "Weekly"<br>4 = "Daily or almost daily"	0= Not at all<br>1= Sometimes, weekly or daily
Tobacco use	"Do you smoke?"<br>"Do you use snuff?"	1= "Not at all"<br>2= "Yes previously but I have quit"<br>3= "Less than once a week"<br>4= "Weekly but not daily"<br>5= "Daily"	0= Not at all or having quit<br>1= Yes
Educational sectors	In which university do you study?<br>In which university of applied siences do you study?	1= "Universities"<br>2= "Universities of applied sciences"	0= Universities<br>1= Universities of applied sciences
Age	Age in years?	In six groups<br>1= 19-22 years<br>2= 23-24 years<br>3= 25-27 years<br>4= 28-30 years<br>5= 31-33 years<br>6= 34 -35<br>In three group<br>1= 19-24<br>2= 25- 30<br>3= 31-35	0= 19-21<br>1= 22 years or older
Gender	Gender<br>1= Male<br>2= Female<br>3= Other	1= Men<br>2= Women	0= Men<br>1= Women
